# Copper impurity of iron raw material contributes to improved cell culture performance

**DOI:** 10.1002/btpr.3251

**Published:** 2022-04-18

**Authors:** Christine Hilde Weiss, Janine Stephanie Caspari, Corinna Merkel, Aline Zimmer

**Affiliations:** ^1^ Upstream R&D Merck Life Science Darmstadt Germany; ^2^ Institute for Organic Chemistry and Biochemistry Technische Universität Darmstadt Darmstadt Germany

**Keywords:** Cell culture medium, copper, iron, low impurity

## Abstract

Cell culture medium (CCM) formulations are chemically defined to reduce lot‐to‐lot variability and complexity of the medium while still providing all essential nutrients supporting cell growth and productivity of various cell lines. However, raw material impurities may still introduce variations and inconsistencies to final CCM formulations. In one of our previous studies (Weiss et al. Biotechnol Prog. 2021;37(4):e3148), we have demonstrated the impact of iron raw material impurity on Chinese hamster ovary (CHO) cell performance and critical quality attributes (CQAs) of recombinant proteins within the Cellvento® 4CHO CCM platform by identifying manganese impurity as the main root cause for improved cell performance and altered glycosylation profiles. This study sought to investigate the impact of iron raw material impurities within another medium platform, namely EX‐CELL® Advanced CHO Fed‐Batch‐Medium. As opposed to previously published results, in this platform, copper instead of manganese impurity present within the used ferric ammonium citrate (FAC) iron source was responsible for an improved cell performance of a CHOZN® cell line and a slight difference in CQAs of the produced recombinant protein. The use of tightly controlled raw material specifications or the use of low impurity iron sources is therefore crucial to minimize the impact of impurities on cell performance in any CCM platform and thereby guarantee consistent and reproducible cell culture processes.

## 1 INTRODUCTION

Cultivation of Chinese hamster ovary (CHO) cells is widely used to produce recombinant proteins for several applications within the pharmaceutical industry,^1,2^ whereas the cell culture medium (CCM) composition is one major factor impacting cell performance and critical quality attributes (CQAs) of the final product.^3^, ^4^, ^5^ In order to reduce lot‐to‐lot variations and the complexity of the CCM, chemically defined media have been developed comprising up to 100 components including amino acids, carbohydrates, vitamins, lipids, inorganic salts and trace elements.^5^, ^6^, ^7^ However, these CCM may still contain variabilities due to trace metal impurities present within raw materials.^8^

Since, on the one hand, iron is an essential compound needed in CCM due to its central role in many cellular processes such as energy metabolism or antioxidant functions,^9^ but on the other hand it can catalyze Fenton reactions resulting in the formation of reactive oxygen species (ROS) due to its redox capability,^10^, ^11^ a previous study of our group investigated the impact of iron raw material and its impurities on CHO cell performance and recombinant protein product quality within the Cellvento® 4CHO medium platform.^12^ Thereby, manganese present as impurity within the iron source was identified as the major contributing factor to an overall improved cell performance and altered glycosylation profile of the recombinant proteins, whereas iron itself showed contrary effects on both parameters.^12^

However, since different chemically defined media vary significantly in their final formulation,^13^ the impact of especially iron raw material impurities may be different for specific CCM platforms. Within this study, the impact of iron raw material impurities on cell performance and product quality (aggregation and glycosylation profile) of a CHOZN® cell line within another medium platform, namely EX‐CELL® Advanced CHO Fed‐Batch‐Medium, was investigated. Results revealed that, contrary to the previously performed study, copper rather than manganese impurity present within the used ferric ammonium citrate (FAC) iron source was the root cause for improved cell performance, whereas also CQAs of the fusion protein seemed to be slightly affected upon copper presence. Altogether, these results reinforce the need for low impurity iron sources in CCM to develop consistent and reproducible cell culture processes independently of the used CCM platform.

## 2 MATERIAL AND METHODS

### 2.1 Cell culture experiments

All raw materials and chemicals used for cell culture experiments were purchased from Merck KGaA, Darmstadt, Germany if not stated otherwise. For fed‐batch cultures, a CHOZN® clone producing a fusion protein was seeded at 2 x10^5^ cells/ml in a working volume of 30 ml in iron‐deficient chemically defined EX‐CELL® Advanced CHO Fed‐Batch‐Medium supplemented either with ferric ammonium citrate A (FAC_A_) or ferric ammonium citrate B (FAC_B_) iron source, coming from two different supply chains, or low trace element impurity FAC (FAC_Synt_), which was synthesized in‐house using a proprietary process, at a final iron concentration of 16 mg/L (mass% of Fe in FAC_A_: 16.7%, mass% of Fe in FAC_B_: 18.0%, mass% of Fe in FAC_Synt_: 18.7%). Additionally, for some conditions, copper (II) sulfate pentahydrate (addition of 21.3 or 22.7 μg/L from a 0.1 g/L stock upon either FAC_B_ or FAC_Synt_ usage, respectively), sodium molybdate (VI) dihydrate (addition of 4.83 μg/L from a 0.1 g/L stock upon FAC_B_ usage), tin (II) chloride dihydrate (0.70 μg/L from a 0.02 g/L stock upon FAC_B_ usage) or a mixture of all three compounds were added to the CCM containing FAC_B_ or FAC_Synt_ to obtain the same concentrations as present upon FAC_A_ usage. The fed‐batch experiments were performed in 50 ml spin tubes (TPP) with vented cap at 37°C, 5% CO_2_, 80% humidity and with an agitation speed of 230 rpm. 5% (v/v) of original glucose‐containing EX‐CELL® Advanced CHO Feed 1 were added on day 3, 5, 7, 10, 12 and 14. Glucose (400 g/L) was fed on demand to up to 6 g/L during the week and up to 10 g/L over the weekend. Viable cell density (VCD) and viability were measured with the Vi‐CELL™XR 2.04 (Beckman Coulter). Glucose, titer, lactate and ammonium concentrations were analyzed with the Cedex Bio HT (Roche) after centrifugation of the sample for 5 min at 4500 rpm (2287 g).

### 2.2 Antibody purification and CQAs analysis

Fusion proteins were purified from the cell culture supernatant on day 10 of the fed‐batch experiments by using protein A PhyTips® (PhyNexus Inc.). Aggregation and glycosylation profiles were analyzed using size exclusion chromatography coupled to an UV detector (SEC‐UV) and ultra‐performance liquid chromatography (UPLC) coupled to a mass spectrometer (UPLC‐MS), respectively, as described elsewhere.^12^, ^14^

### 2.3 Iron source characterization

The detection and quantification of trace elements within iron sources was performed either by a semiquantitative elemental screening method using inductively coupled plasma mass spectrometry (ICP‐MS) or by a quantification with external calibration using high resolution (HR)‐ICP‐MS as described elsewhere.^12^

### 2.4 Statistical analysis

Data are expressed as means ± standard deviation (SD) of biological replicates, whereas the graphical analysis was performed with GraphPad Prism 9 software (GraphPad Software Inc.).

## 3 RESULTS

### 3.1 Effect of two iron sources, FAC_A_ and FAC_B_ , coming from different supply chains on cell performance and CQAs within EX‐CELL® Advanced CHO Fed‐Batch‐Medium

**FIGURE 1:**
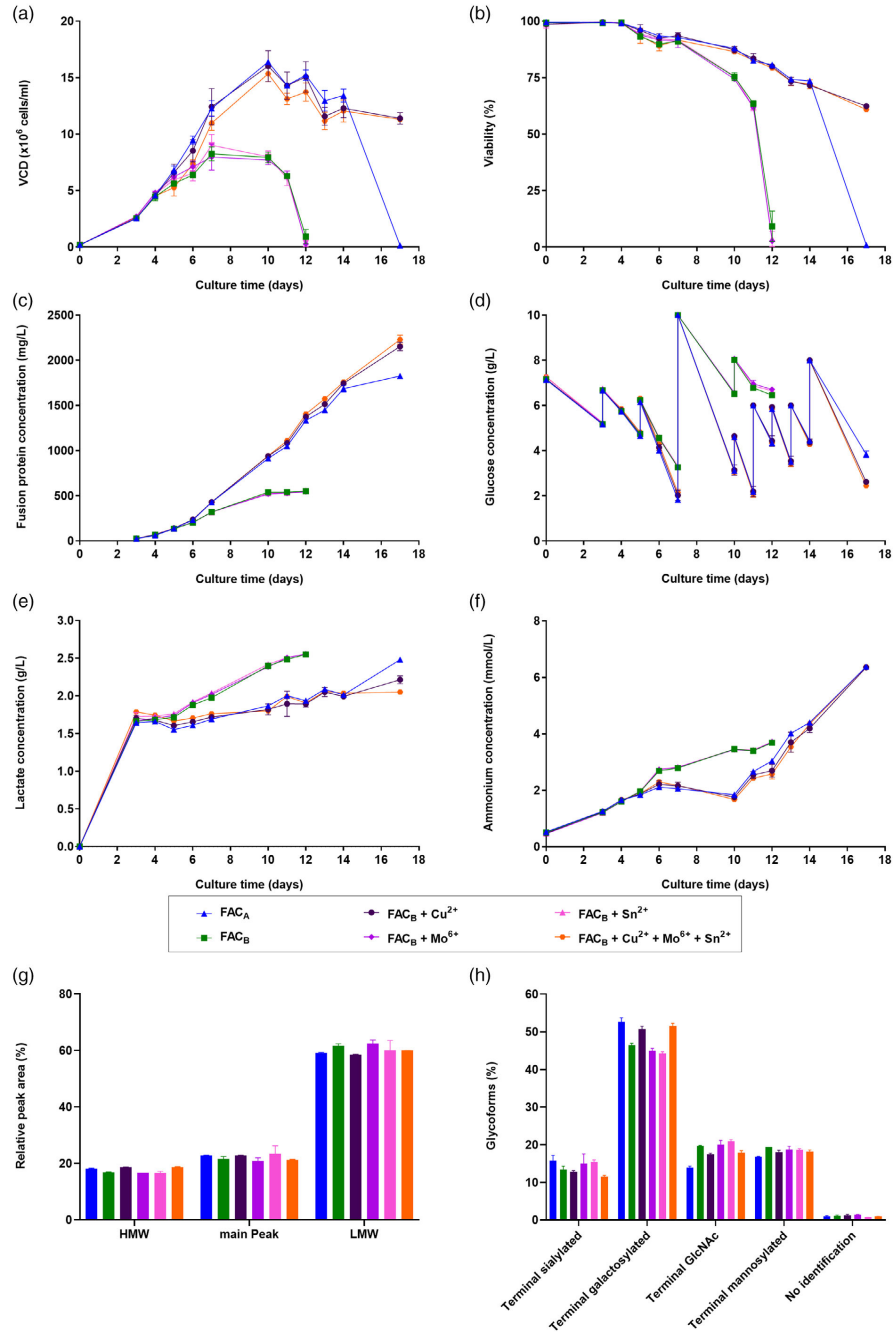
Effect of iron sources FAC_A_, FAC_B_ and FAC_B_ supplemented with either copper, molybdate, tin or a combination of them on cell performance and CQAs of fusion protein. CHOZN® cells were cultivated in medium supplemented with either FAC_A_ or FAC_B_ iron source. Additionally, four further conditions were prepared, where either copper, molybdate, tin or all three elements were added to FAC_B_ to achieve the exact same respective concentrations as present in FAC_A_. (a) VCD in x10^6^ cells/ml. (b) Viability in %. (c) Fusion protein concentration in mg/L. (d) Glucose concentration in g/L. (e) Lactate concentration in g/L. (f) Ammonium concentration in mmol/L. (g) HMW, main peak and LMW level of fusion protein in %. (h) N‐glycosylation forms (terminal sialylated, terminal galactosylated, terminal GlcNAc and terminal mannosylated) of fusion protein in %. Data are mean ± SD of either four (a–f) or two (g,h) replicates

Following the recent findings within Cellvento® 4CHO and 4Feed fed‐batch platform,^12^ it was of great interest to investigate whether the usage of two different FAC iron sources coming from different supply chains might also cause an altered cell performance and/or altered CQAs when supplemented to another chemically defined medium platform. Therefore, two FAC iron sources, namely FAC_A_ and FAC_B_, were added to iron‐deficient EX‐CELL® Advanced CHO Fed‐Batch‐Medium at a final iron concentration of 16 mg/L and a small‐scale fed‐batch experiment was performed. Results (Figure 1, blue and green curves) revealed a significantly improved VCD upon FAC_A_ usage with a nearly two‐times higher maximal VCD compared to FAC_B_ (Figure 1a), as well as a prolonged cell culture viability throughout the fed‐batch experiment (Figure 1b). A three‐times higher final titer of the fusion protein was detected upon FAC_A_ usage compared to FAC_B_ iron source (Figure 1c). Glucose consumption rate was higher during the second half of the fed‐batch process upon FAC_A_ usage compared to FAC_B_ (Figure 1d) and a faster increase in lactate and ammonium accumulation within cell culture supernatant was observed for FAC_B_ (Figure 1e,f). Investigation of the aggregation profile showed no difference upon usage of either FAC_A_ or FAC_B_ (Figure 1g), however, a slightly different glycosylation profile of the fusion protein was detected when comparing the two iron sources (Figure 1h). Similarly to what was already observed within the Cellvento® 4CHO platform,^12^ manganese impurity level present within the used iron raw materials was hypothesized as root cause for the observed differences. However, even though the manganese impurity level showed slight variations within the used iron sources (FAC_A_: 56 μg Mn/g FAC and FAC_B_: 38 μg Mn/g FAC), usage of an aligned manganese concentration in both iron sources (FAC_A_ and FAC_B_) did not restore the cell performance to a similar level. Only the observed glycosylation profile differences of the fusion protein detected upon usage of either FAC_A_ or FAC_B_ were reduced after alignment of the manganese level (data not shown).

Since the manganese impurity present within both iron sources contributed in a similarly high manner to the overall total manganese concentration in the CCM (>97%), the results thus suggested that impurities other than manganese might have impacted the cell performance within the EX‐CELL® Advanced CHO Fed‐Batch‐Medium platform, especially since other trace elements besides manganese are known to affect cell performance.^8^, ^15^

### 3.2 Analysis of elemental impurities in FAC_A_ and FAC_B_

A full characterization of elemental impurities present within both iron sources (FAC_A_ and FAC_B_) was performed by semiquantitative ICP‐MS or quantitative HR‐ICP‐MS analysis. Table 1 summarizes all obtained elemental impurities with values above 4 μg/g for at least one iron source. Whereas some elements such as potassium (K), zinc (Zn) and gallium (Ga) were only detected in FAC_B_, other elements were found in both iron sources. Higher trace element impurity levels for manganese (Mn), nickel (Ni), copper (Cu), molybdate (Mo) and tin (Sn) were observed in FAC_A_, whereas magnesium (Mg), aluminum (Al), calcium (Ca), titanium (Ti) and vanadium (V) showed higher levels in FAC_B_. Similar impurity levels were detected for chromium (Cr) and cobalt (Co).

**TABLE 1:** Impurity profile of iron sources FAC_A_, FAC_B_ and FAC_Synt_

μg/g	Fe	Mg	Al	K^a^	Ca^a^	Ti^a^	V^a^	Cr	Mn	Co	Ni	Cu	Zn	Ga	Mo	Sn
FAC_A_	167,000	48	34	<5.0	100	32	60	7.5	56	20	50	60	<1.0	<5.0	21	4.5
FAC_B_	180,000	150	110	5.3	200	120	140	8.5	38	21	40	4	190	10	1.2	0.75
FAC_Synt_	187,000	<1.0	<2.0	5.3	26.0	<1.0	<0.1	1.8	<0.2	<0.1	<0.2	<0.1	2.0	<0.5	<0.5	<0.5

*Notes*: Only elements showing values above 4 μg/g raw material for at least one iron source are presented, whereby the impurity characterization was done by semiquantitative ICP‐MS if not stated otherwise. Fe, iron; Mg, magnesium; Al, aluminum; K, potassium; Ca, calcium; Ti, titanium; V, vanadium; Cr, chromium; Mn, manganese; Co, cobalt; Ni, nickel; Cu, copper; Zn, zinc; Ga, gallium; Mo, molybdate; Sn, tin

^a^ Quantitative values gained with HR‐ICP‐MS, whereby the obtained calibration curves yielded a correlation coefficient of at least >0.995

The next step then focused on identifying those trace elements with significant different level of abundance in both tested FAC iron sources and investigating their potential on improving cell performance. Considering the smaller number of elements showing a higher impurity level in FAC_A_ compared to FAC_B_ and the significant higher impurity level of Cu, Mo and Sn in FAC_A_ compared to FAC_B_ (more than five times higher), initial investigations only focused on the three mentioned elements.

### 3.3 Effect of copper, molybdate and tin on cell performance and CQAs

To investigate the impact of Cu, Mo and Sn present as impurity in FAC_A_ on CHOZN® cell performance in EX‐CELL® Advanced CHO Fed‐Batch‐Medium, a small‐scale fed‐batch experiment in spin tubes was performed by comparing FAC_A_ and FAC_B_ iron source at a final iron concentration of 16 mg/L, whereby the impurity levels of Cu, Mo and Sn were also individually adjusted for FAC_B_ to the exact same level as present in FAC_A_. Additionally, the combinatory effect of all three trace elements on CHOZN® cell performance was studied upon supplementation to FAC_B_. As shown in Figure 1, cell performance upon FAC_B_ usage was significantly affected upon trace element supplementation. Whereas the addition of only molybdate or tin to FAC_B_ resulted in a similar low cell growth compared to FAC_B_ alone with an average maximum VCD of 8.49 ×10^6^ cells/ml detected on day 7, supplementation of copper to FAC_B_ led to a similar VCD profile as obtained for FAC_A_ with an average maximum VCD of 15.69 ×10^6^ cells/ml detected on day 10 (Figure 1a). Usage of either FAC_B_ alone or FAC_B_ supplemented with either molybdate or tin resulted in a faster decline of cell culture viability compared to the other tested conditions. Addition of copper to FAC_B_ maintained cell culture viability above 60% until the end of the fed‐batch experiment compared to the already detected cell death on day 17 upon FAC_A_ usage (Figure 1b). The addition of copper to FAC_B_ iron source also led to a significantly higher final titer compared to only FAC_B_ or FAC_B_ supplemented with only molybdate or tin with final fusion protein concentrations of around 2191.3 mg/L compared to only roughly 549.3 mg/L, respectively. Interestingly, an even higher final titer was observed for FAC_B_ supplemented with copper (average titer of 2191.3 mg/L) compared to FAC_A_ iron source (1826.3 mg/L) on day 17 (Figure 1c). Glucose consumption rate was similar throughout the first seven days of the fed‐batch process for any tested condition, whereas copper‐containing conditions consumed more glucose in the following days, resulting from a higher VCD and viability compared to the other conditions (Figure 1d). A lower lactate accumulation within cell culture supernatant during the fed‐batch experiment was detected upon FAC_A_ and FAC_B_ supplemented with copper compared to the other tested conditions (Figure 1e), whereas a faster increase in ammonium accumulation was detected upon FAC_B_ and FAC_B_ supplemented with either molybdate or tin (Figure 1f). The detected absolute differences in high molecular weight (HMW), low molecular weight (LMW) and main peak level for fusion protein were less than 2.2, 4.1 and 2.7%, respectively, for all tested conditions (Figure 1g). Fusion protein glycosylation results revealed slightly higher levels of terminal galactosylated species and slightly lower terminal GlcNAc levels upon FAC_A_ and FAC_B_ usage supplemented with copper compared to the other tested conditions with an average absolute altered level of 6.4 and 3.8%, respectively. This difference, however, might have been caused by a still higher cell culture viability for those conditions on day 10. The direct impact of copper on these glycoforms would require further investigation that is beyond the scope of this note. No significant copper‐, molybdate‐ or tin‐related effect on terminal sialylated or terminal mannosylated species was observed (Figure 1h).

Overall, the results indicate that, contrary to the observations made within Cellvento® 4CHO platform, copper instead of manganese impurity present within the FAC_A_ iron source contributed to an overall improved cell performance of the tested CHOZN® cell line in EX‐CELL® Advanced CHO Fed‐Batch‐Medium as well as slightly altered CQAs. This difference was especially observed due to the comparison of two different iron sources (coming from two supply chains) having different copper impurity levels, whereby a comparison of copper impurity levels present within several lots of the same iron source resulted in a standard deviation of ±8.51 μg/g (FAC_A_) and ±3.13 μg/g (FAC_B_). The copper impurities present in FAC_A_ and FAC_B_ thus contributed either to more than 63% or less than 26% to the final medium formulation, respectively.

### 3.4 Effect of low impurity FAC iron source (FAC_Synt_ ) on cell performance

**FIGURE 2:**
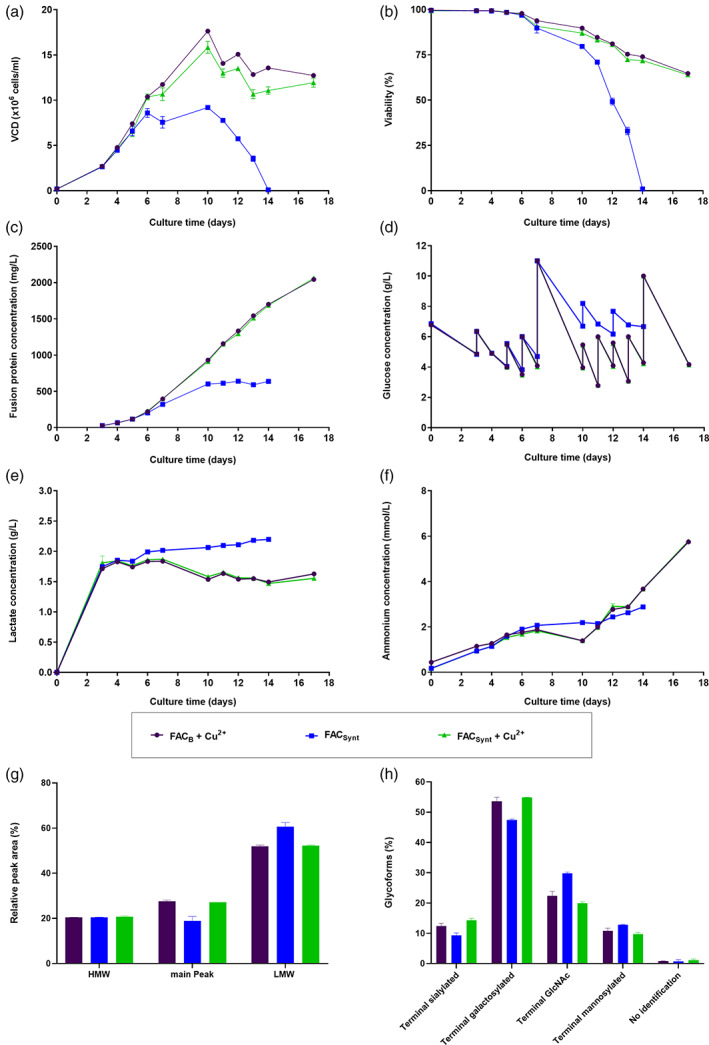
Effect of low impurity iron source FAC_Synt_ and FAC_Synt_ supplemented with copper on cell performance and CQAs of fusion protein compared to FAC_B_ supplemented with copper. CHOZN® cells were cultivated in medium supplemented with either FAC_B_ + Cu^2+^, FAC_Synt_ or FAC_Synt_ + Cu^2+^. (a) VCD in x10^6^ cells/ml. (b) Viability in %. (c) Fusion protein concentration in mg/L. (d) Glucose concentration in g/L. (e) Lactate concentration in g/L. (f) Ammonium concentration in mmol/L. (g) HMW, main peak and LMW level of fusion protein in %. (h) N‐glycosylation forms (terminal sialylated, terminal galactosylated, terminal GlcNAc and terminal mannosylated) of fusion protein in %. Data are mean ± SD of either three (a–f) or two (g,h) replicates

Although the addition of copper to FAC_B_ iron source to match the same copper concentration as present within FAC_A_ improved the cell performance significantly, there was still a difference observed in cell performance compared to FAC_A_ that may be due to further impurities. Other trace elements that have been either reported in literature for their positive effect on cell growth, viability or titer were for instance zinc and vanadium,^16^, ^17^ even though an excess of vanadium was also reported to cause a cytotoxicity within CHO cells.^18^, ^19^ In order to investigate whether impurities present within FAC_A_ might have led to a toxic effect and thus might have caused a faster decline in cell culture viability compared to FAC_B_ supplemented with copper, an in‐house synthesized low impurity FAC iron source (FAC_Synt_; impurity profile presented in Table 1) was used and compared to the previously used FAC_B_ + Cu^2+^ condition. The setup of this small‐scale fed‐batch experiment was similar to previous experiments and relied on the supplementation of the new iron sources to iron‐deficient EX‐CELL® Advanced CHO Fed‐Batch‐Medium at a final iron concentration of 16 mg Fe/L. Additionally, due to the previous findings, one further condition was tested, for which copper was added to FAC_Synt_ to match the exact same concentration as present in FAC_B_ + Cu^2+^. Results revealed a similar cell growth curve upon FAC_Synt_ usage supplemented with copper compared to FAC_B_ + Cu^2+^, whereas usage of low copper‐containing FAC_Synt_ alone resulted in a lower maximal VCD (Figure 2a). Cell culture viability was maintained again above 60% until the end of the fed‐batch for copper‐containing conditions, whereas usage of FAC_Synt_ led to a detected cell death on day 14 (Figure 2b). A similar final fusion protein concentration of around 2053.3 mg/L was detected for FAC_B_ and FAC_Synt_, both supplemented with copper, which was more than three‐times higher compared to FAC_Synt_ (Figure 2c). Glucose consumption rate was similar throughout the first seven days of the fed‐batch process for any tested condition, whereas the glucose consumption rate was higher for copper‐containing conditions in the following days as a result of increased VCD and viability compared to FAC_Synt_ (Figure 2d). Usage of FAC_Synt_ alone led to a higher lactate and a similar ammonium accumulation within cell culture supernatant during the fed‐batch experiment compared to copper‐supplemented FAC_B_ and FAC_Synt_ (Figure 2e,f). The detected absolute difference in HMW level for fusion protein was less than 0.4%, whereby a reduced main peak level was observed upon FAC_Synt_ usage compared to copper‐containing conditions, which was inversely correlated to the LMW level (Figure 2g). Fusion protein glycosylation results revealed similar profiles for FAC_B_ and FAC_Synt_ when supplemented with copper, whereby the absolute differences were less than 2.4% for all presented glycosylation forms. Usage of only FAC_Synt_ led to a reduced terminal galactosylation level and increased terminal GlcNAc level compared to the copper‐supplemented conditions similarly to what was already observed upon usage of FAC_B_ only (Figure 2h). On the one hand, these results confirm the positive contribution of copper to overall cell performance thus also affecting glycosylation profile of the recombinant protein. On the other hand, the results suggest that impurities present within some iron sources (FAC_A_) might be detrimental to cell culture and responsible for the decreased cell viability and lower final titer observed at the end of the fed‐batch.

Overall, the results thus reinforce the impact of iron raw material impurities on cell culture performance and the need for low impurity iron sources in CCM formulations to decouple the effects of iron and other elements in cell culture processes. This is particularly relevant for media development scientists since impurities can have either a positive effect on performance and CQAs or an unwanted toxic effect, even at very low concentrations.

## 4 DISCUSSION

Iron raw material impurities, especially manganese, were already identified as major contributors for altered cell performance and protein glycosylation level within Cellvento® 4CHO CCM platform.^12^ This study sought to investigate the impact of iron raw material impurity on cell performance and CQAs of a CHOZN® clone within another CCM platform, namely EX‐CELL® Advanced CHO Fed‐Batch‐Medium by performing small‐scale fed‐batch experiments. Thereby, copper rather than manganese impurity was identified to significantly contribute to an improved cell performance with a reduced lactate accumulation within cell culture supernatant, whereas a slight impact of copper impurity on CQAs of the fusion protein was observed.

A positive effect of copper on cell growth and titer upon supplementation to CCM was already reported in literature,^15^, ^20^ whereby a more efficient energy production via the oxidative phosphorylation pathway resulting in a reduced overall lactate accumulation within cell culture was suggested.^15^, ^20^, ^21^, ^22^ In contrast, a copper limitation was reported to favor glycolysis over citric acid cycle as the energy generation route leading to a higher lactate accumulation within cell culture.^21^, ^22^ This hypothesized copper‐related difference in energy metabolism was proposed to involve copper‐dependent enzymes involved in the mitochondrial electron transport chain such as cytochrome c oxidase, an enzyme responsible for the last step of the electron transfer within the mitochondrial respiratory chain.^21^, ^23^ Within this study, it is thus likely that an enhanced oxidative capacity of the cells was induced either by an increased protein expression level and/or activity of cytochrome c oxidase upon copper presence as reported in several studies,^24^, ^25^, ^26^, ^27^ and thereby leading to an overall improved cell performance and thus to a reduced lactate accumulation within cell culture.

Alternatively, since copper is a known cofactor for the copper/zinc superoxide dismutase 1 (SOD1), an enzyme involved in protecting the cells from reactive species produced during cellular respiration,^28^, ^29^, ^30^ copper might have increased the cellular antioxidant defense capacity in a similar manner as it was suggested for manganese,^12^ leading to an increased cell growth and fusion protein production. SOD1 is preventing cellular lipid peroxidation and deoxyribonucleic acid (DNA) damages by dismutating superoxide anions and is thus an essential enzyme of the cellular antioxidant defense system.^30^

Although copper impurity significantly improved the cell performance, the presence of further raw material impurities within FAC_A_ was suggested to cause a faster decline in cell culture viability and a reduced final titer when comparing to low impurity FAC_Synt_ iron source. Since iron sources used for cell culture processes are usually obtained from poorly characterized starting materials (iron ores used by other industries), and are further processed by applying strong acids such as sulfuric acid leading to the final raw material used in CCM (ferrous sulfate),^31^, ^32^, ^33^, ^34^, ^35^, ^36^ raw material impurities are likely introduced and batch‐to‐batch variability is expected due to this heterogeneity in starting materials.^37^, ^38^ Further root causes for impurities in iron sources may result from the use of low‐grade solvents or contaminated or leaching packaging materials during the manufacturing process.^37^, ^38^ To ensure process consistency for biologics, tight specifications should be applied for the release of such high risk raw materials and should be based on sensitive analytical methods such as quantitative ICP‐MS.^39^, ^40^ Additional assessments should be performed to identify other risky raw materials in formulations and studies have to be performed to generate knowledge about the possible impact of their impurities on the final cell culture process performance.^37^, ^39^ In case the raw material manufacturing process or the inherent heterogeneity of starting source materials does not enable a tight control of the specifications and thus does not support a constant quality, new processes or supply chains should be established to guarantee the constant supply of low impurity raw materials with drastically reduced risks of variability.

## 5 CONCLUSION

Overall, this study describes the impact of iron raw material impurity, in this case of copper impurity, on cell culture performance in EX‐CELL® Advanced CHO Fed‐Batch‐Medium leading to an overall improved cell growth, titer and prolonged viability, whereas CQAs of the tested fusion protein were only slightly affected upon copper presence. This study further highlights the need for low impurity iron sources in CCM formulations in order to decouple the effects of iron and other contaminating trace elements thereby allowing the development of consistent and stable cell culture processes without unwanted impurity‐related positive or even toxic effects.

## CONFLICT OF INTEREST

All authors are employees of Merck KGaA, Germany.

## AUTHOR CONTRIBUTIONS

**Christine Hilde Weiss:** Investigation (equal); validation (equal); visualization (equal); writing – original draft (equal). **Janine Stephanie Caspari:** Investigation (equal); validation (equal). **Corinna Merkel:** Supervision (equal); validation (equal). **Aline Zimmer:** Supervision (equal); validation (equal).

### PEER REVIEW

The peer review history for this article is available at https://publons.com/publon/10.1002/btpr.3251.

## Data Availability

All data are contained within the manuscript.

## References

[1] Grilo AL , Mantalaris A . The increasingly human and profitable monoclonal antibody market. Trends Biotechnol. 2019;37(1):9‐16. doi:10.1016/j.tibtech.2018.05.014 29945725

[2] Wurm FM . Production of recombinant protein therapeutics in cultivated mammalian cells. Nat Biotechnol. 2004;22(11):1393‐1398. doi:10.1038/nbt1026 15529164

[3] Brühlmann D , Jordan M , Hemberger J , Sauer M , Stettler M , Broly H . Tailoring recombinant protein quality by rational media design. Biotechnol Prog. 2015;31(3):615‐629. doi:10.1002/btpr.2089 25864704

[4] Huang Y‐M , Hu W , Rustandi E , Chang K , Yusuf‐Makagiansar H , Ryll T . Maximizing productivity of CHO cell‐based fed‐batch culture using chemically defined media conditions and typical manufacturing equipment. Biotechnol Prog. 2010;26(5):1400‐1410. doi:10.1002/btpr.436 20945494

[5] Li F , Vijayasankaran N , Shen AY , Kiss R , Amanullah A . Cell culture processes for monoclonal antibody production. MAbs. 2010;2(5):466‐479. doi:10.4161/mabs.2.5.12720 20622510 PMC2958569

[6] Calvet A , Ryder AG . Monitoring cell culture media degradation using surface enhanced Raman scattering (SERS) spectroscopy. Anal Chim Acta. 2014;840:58‐67. doi:10.1016/j.aca.2014.06.021 25086894

[7] Jordan M , Stettler M , Broly H . Will we ever find a perfect medium for mammalian cell culture? Pharm Bioprocess. 2013;1(5):411‐413. doi:10.4155/pbp.13.50

[8] Graham RJ , Bhatia H , Yoon S . Consequences of trace metal variability and supplementation on Chinese hamster ovary (CHO) cell culture performance: a review of key mechanisms and considerations. Biotechnol Bioeng. 2019;116(12):3446‐3456. doi:10.1002/bit.27140 31403183

[9] Dlouhy AC , Outten CE . The iron Metallome in eukaryotic organisms. Met Ions Life Sci. 2013;12:241‐278. doi:10.1007/978-94-007-5561-1_8 23595675 PMC3924584

[10] Anderson GJ , Vulpe CD . Mammalian iron transport. Cell Mol Life Sci. 2009;66(20):3241‐3261. doi:10.1007/s00018-009-0051-1 19484405 PMC11115736

[11] Dev S , Babitt JL . Overview of iron metabolism in health and disease. Hemodial Int. 2017;21(Suppl 1):S6‐S20. doi:10.1111/hdi.12542 28296010 PMC5977983

[12] Weiss CH , Merkel C , Zimmer A . Impact of iron raw materials and their impurities on CHO metabolism and recombinant protein product quality. Biotechnol Prog. 2021;37(4):e3148. doi:10.1002/btpr.3148 33742789 PMC8459231

[13] Reinhart D , Damjanovic L , Kaisermayer C , Kunert R . Benchmarking of commercially available CHO cell culture media for antibody production. Appl Microbiol Biotechnol. 2015;99(11):4645‐4657. doi:10.1007/s00253-015-6514-4 25846330 PMC4435641

[14] Ehret J , Zimmermann M , Eichhorn T , Zimmer A . Impact of cell culture media additives on IgG glycosylation produced in Chinese hamster ovary cells. Biotechnol Bioeng. 2019;116(4):816‐830. doi:10.1002/bit.26904 30552760 PMC6590254

[15] Yuk IH , Russell S , Tang Y , et al. Effects of copper on CHO cells: cellular requirements and product quality considerations. Biotechnol Prog. 2015;31(1):226‐238. doi:10.1002/btpr.2004 25311542

[16] Capella Roca B , Alarcón Miguez A , Keenan J , et al. Zinc supplementation increases protein titer of recombinant CHO cells. Cytotechnology. 2019;71(5):915‐924. doi:10.1007/s10616-019-00334-1 31396753 PMC6787129

[17] McGrew JT . Cell culture performance with vanadate. US Patent 6974681B1. 2005.

[18] Zwolak I , Gołębiowska D . Protective activity of pyruvate against vanadium‐dependent cytotoxicity in Chinese hamster ovary (CHO‐K1) cells. Toxicol Ind Health. 2018;34(5):283‐292. doi:10.1177/0748233718754979 29529943

[19] Zwolak I . Increased cytotoxicity of vanadium to CHO‐K1 cells in the presence of inorganic selenium. Bull Environ Contam Toxicol. 2015;95(5):593‐598. doi:10.1007/s00128-015-1615-4 26201834 PMC4608973

[20] Xu S , Hoshan L , Chen H . Improving lactate metabolism in an intensified CHO culture process: productivity and product quality considerations. Bioprocess Biosyst Eng. 2016;39(11):1689‐1702. doi:10.1007/s00449-016-1644-3 27324235

[21] Luo J , Vijayasankaran N , Autsen J , et al. Comparative metabolite analysis to understand lactate metabolism shift in Chinese hamster ovary cell culture process. Biotechnol Bioeng. 2012;109(1):146‐156. doi:10.1002/bit.23291 21964570

[22] Nargund S , Qiu J , Goudar CT . Elucidating the role of copper in CHO cell energy metabolism using (13)C metabolic flux analysis. Biotechnol Prog. 2015;31(5):1179‐1186. doi:10.1002/btpr.2131 26097228

[23] Horn D , Barrientos A . Mitochondrial copper metabolism and delivery to cytochromec oxidase. IUBMB Life. 2008;60(7):421‐429. doi:10.1002/iub.50 18459161 PMC2864105

[24] Zeng H , Saari JT , Johnson WT . Copper deficiency decreases complex IV but not complex I, II, III, or V in the mitochondrial respiratory chain in rat heart. J Nutr. 2007;137(1):14‐18. doi:10.1093/jn/137.1.14 17182794

[25] Kang S , Xiao G , Ren D , et al. Proteomics analysis of altered cellular metabolism induced by insufficient copper level. J Biotechnol. 2014;189:15‐26. doi:10.1016/j.jbiotec.2014.08.001 25150618

[26] Yuk IH , Zhang JD , Ebeling M , et al. Effects of copper on CHO cells: insights from gene expression analyses. Am Inst Chem Eng Biotechnol Prog. 2014;30(2):429‐442. doi:10.1002/btpr.1868 24403277

[27] Gybina AA , Prohaska JR . Copper deficiency results in AMP‐activated protein kinase activation and acetylCoA carboxylase phosphorylation in rat cerebellum. Brain Res. 2008;1204:69‐76. doi:10.1016/j.brainres.2008.01.087 18339363 PMC2390879

[28] Percival SS , Harris ED . Regulation of cu,Zn superoxide dismutase with copper. Caeruloplasmin maintains levels of functional enzyme activity during differentiation of K562 cells. Biochem J. 1991;274(Pt 1):153‐158. doi:10.1042/BJ2740153 1900417 PMC1150191

[29] Boyd SD , Ullrich MS , Skopp A , Winkler DD . Copper sources for Sod1 activation. Antioxidants. 2020;9(6):500. doi:10.3390/antiox9060500 PMC7346115 32517371

[30] M Fetherolf M , Boyd SD , Winkler DD , Winge DR . Oxygen‐dependent activation of Cu, Zn‐superoxide dismutase‐1. Metallomics. 2017;9(8):1047‐1059. doi:10.1039/C6MT00298F 28686251

[31] Mattia FJ , Sakler SA . Process for producing ferrous sulfate. US Patent 3760069A. 1973.

[32] Gates KW , Roberts SN . Process for the production of ferrous sulphate monohydrate. US Patent 0052106A1. 2013.

[33] Wang Y , Yan J , Wang X , Li Z . Synthesis of ferric ammonium citrate. Huagong Jishu Yu Kaifa. 2014;43(7):25‐27.

[34] Zhang L , Dong X , Shu Y , Pang C , Liu Y . Preparation of ammonium citrate by iron mud. Shenyang Huagong Xueyuan Xuebao. 2009;23(2):109‐113.

[35] Shan C , Xin W , Siqin Z . Method for synthesizing ammonium ferric citrate. CN Patent 108456137A 2018.

[36] Kruse HJ , Mounce HC . Crystalline ferric ammonium citrate compounds. US Patent 2644828A. 1953.

[37] Grinnell C , Bareford L , Matthews TE , Brantley T , Moore B , Kolwyck D . Elemental metal variance in cell culture raw materials for process risk profiling. Biotechnol Prog. 2020;36(5):e3004. doi:10.1002/btpr.3004 32309907

[38] Challener CA . Biopharma takes on raw material variability. BioPharm Int. 2016;29(3):20‐24.

[39] Dickens J , Khattak S , Matthews TE , Kolwyck D , Wiltberger K . Biopharmaceutical raw material variation and control. Curr Opin Chem Eng. 2018;22:236‐243. doi:10.1016/j.coche.2018.10.007

[40] Mohammad A , Agarabi C , Rogstad S , et al. An ICP‐MS platform for metal content assessment of cell culture media and evaluation of spikes in metal concentration on the quality of an IgG3:κ monoclonal antibody during production. J Pharm Biomed Anal. 2019;162:91‐100. doi:10.1016/j.jpba.2018.09.008 30227357

